# Production of low sulfur diesel fuel through ultrasonic catalytic oxidative route using novel mixed oxides nanocomposites assisted by solvent extraction

**DOI:** 10.1038/s41598-026-39220-0

**Published:** 2026-04-10

**Authors:** Asmaa I. Zahran, Esraa M. El-Fawal, Ahmed M. A. El Naggar, Taha F. Hassanein

**Affiliations:** 1 Refining Department, Egyptian Petroleum Research Institute, Nasr City, 11727 Cairo Egypt; 2 Analysis and Evaluation Department, Egyptian Petroleum Research Institute, Nasr City, 11727 Cairo Egypt; 3 Chemistry Department, Faculty of Science, Capital University (Formerly Helwan University), Helwan, 11795 Cairo Egypt

**Keywords:** Diesel fuel, Oxidative desulfurization, Fe-based catalysts, Ultrasound cavitation, Hydrogen peroxide, Chemistry, Environmental sciences, Materials science

## Abstract

This study presents the development of Fe–Co–Ni mixed-metals oxide composites that were synthesized via chemical precipitation method at different reaction times (05–8 h). These composites were subsequently utilized to achieve deep sulfur removal from diesel fuel through ultrasoumd-assisted oxidative desulfurization (UAOD). Among those composites, the obtained composite at a synthesis time of 0.5 h emerged as the most efficient catalyst, achieving nearly 60% sulfur removal under optimized operating conditions (60 °C, 90 min as reaction time, catalyst dose of 10 g/L and a 1:1 H_2_O_2_-to-feed ratio). A subsequent sulfur removal of 89% was achieved through post-treatment with a DMF–acetonitrile solvent mixture (4:1 solvent-to-feed ratio), validating the synergy between catalytic oxidation and solvent extraction. The exceptional performance by this Fe–Co–Ni composite was further amplified by a structure modification through production of core–shell composite using a layer of polystyrene. This composite achieved reduction of sulfur content to 920 ppm (from 21,700 ppm in feedstock) which represented a sulfur removal percentage of approximately 96%. Therefore, the introduced approach can be considered for scalable and energy-efficient processes for producing low-sulfur diesel, aligning with global environmental demands.

**Supplementary Information:**

The online version contains supplementary material available at 10.1038/s41598-026-39220-0.

## Introduction

The escalating demand for ultra-clean diesel fuels, driven by stringent environmental regulations, necessitates the development of efficient desulfurization technologies. Traditional hydrodesulfurization (HDS) processes often fall short in removing refractory sulfur compounds such as dibenzothiophene (DBT) and its derivatives, prompting the exploration of alternative methods. Among these, catalytic oxidative desulfurization (Cat-ODS) has emerged as a promising approach due to its ability to operate under milder conditions and achieve higher sulfur removal efficiencies^1,2^. Therefore, the worldwide environmental regulations have been issued not only to control the sulfur content but also, enforced petroleum processing industry for production of ultra-low sulfur diesel (ULSD) with the content of (less than 8 ppm) sulfur for diesel and gasoline, respectively^2–5^.

Hydrodesulfurization (HDS), a widely utilized conventional desulfurization technology, remains the primary industrial method for removing sulfur compounds such as sulfides, disulfides, thiols, and mercaptans^6^. However, the HDS process requires stringent operating conditions, including high temperatures, high pressures, and the use of costly hydrogen gas and catalysts. These catalysts must possess exceptional chemical and thermal stability to withstand such severe conditions. Despite its effectiveness, these limitations hinder HDS from achieving the deep desulfurization necessary for removing refractory aromatic sulfur compounds^7^. To address the challenges associated with HDS, oxidative desulfurization (ODS) has emerged as an efficient and straightforward alternative in recent years^8^. ODS offers significant advantages for industrial applications, as it operates under mild conditions such as low temperatures and low pressures. Furthermore, ODS can effectively oxidize refractory sulfur compounds into polar derivatives, which can then be easily removed through solvent extraction techniques^9,10^.

The choice of oxidant is a critical factor influencing ODS efficiency. Air and molecular oxygen (O₂) are inexpensive and environmentally benign, but their weak oxidizing ability requires elevated temperatures and longer reaction times, limiting industrial applicability. In contrast, hydrogen peroxide (H₂O₂) is widely favored due to its high oxidizing potential, environmental compatibility, and ability to generate reactive oxygen species (ROS) such as •OH and HO₂•, which selectively oxidize refractory sulfur compounds to sulfones under mild conditions. Peracetic acid (PAA) has also been investigated as a strong oxidant, capable of rapidly oxidizing sulfur compounds; however, its corrosive nature, safety risks, and handling difficulties restrict its widespread application. Comparative studies confirm that H₂O₂ provides the best balance of reactivity, safety, and ease of operation, making it the most practical oxidant for ODS processes^10,11^.

Metal particles smaller than 100 nm in primary particle diameter are generally considered “nanoparticles”. Such metal nanoparticles often exhibit very interesting electronic, magnetic, optical, and chemical properties. For example, their high surface-to-volume ratios have large fractions of metal atoms at surface available for catalysis .In the case of cobalt nanoparticles, they are expected to possess exceptionally high-density magnetic property, sintering reactivity, hardness levels, and excellent impact resistance properties^12–14^. Many studies on synthesis and magnetic properties of nanoscale metal particles and composites have been reported^15,16^. But, so far, in the literature there is no work reporting on synthesis of cobalt nanoparticles by thermal decomposition reaction using cobalt coordination compounds. The preparation of metal particles by thermal decomposition of complexes becomes increasingly important mainly due to the easy control of process conditions, particle size, particle crystal structure, and purity^17–19^.

Transition metal-based catalysts, particularly those incorporating cobalt (Co), iron (Fe), and nickel (Ni), have demonstrated significant potential in enhancing the Cat-ODS process^20^. For instance, studies have shown that silica-supported iron oxide catalysts doped with cobalt and nickel can effectively oxidize sulfur compounds in model diesel fuels. Characterization techniques such as X-ray diffraction (XRD) and scanning electron microscopy (SEM) revealed that these catalysts possess amorphous structures with well-dispersed active sites, contributing to their high catalytic performance^21^. Similarly, the synthesis and characterization of Co/Fe-γAl_2_O_3_ catalysts have been investigated for their efficacy in oxidative desulfurization. These studies employed various analytical methods to elucidate the physicochemical properties of the catalysts, providing insights into their activity and stability under reaction conditions. Furthermore, the application of AFe_2_O_4_ (A: Ni, Co, Mg)–silica nanocomposites in the removal of dibenzothiophene (DBT) through adsorption processes has been explored^22^. Kinetic and isotherm analyses indicated that these nanocomposites exhibit high adsorption capacities, making them suitable candidates for desulfurization application^23^.

Ultrasonic-assisted oxidative desulfurization (UAOD) represents a cutting-edge and environmentally friendly technique, leveraging the synergistic effects of ultrasonic waves and oxidative processes. The action mechanism of ultrasonic waves facilitates the formation of fine emulsions and cavitation bubbles, which generate localized high temperatures and pressures, significantly enhancing reaction rates and desulfurization efficiency. This method also promotes intensified mixing between immiscible phases, resulting in reduced solvent consumption, time, and energy requirements. Despite its promising potential, the combined application of ultrasonic treatment and oxidation for producing clean fuels remains underexplored^24–27^^.^

The novelty of this study lies in the innovative integration of ultrasonic treatment with efficient catalytic oxidation to enhance desulfurization efficiency of real diesel fuel. Unlike conventional oxidative desulfurization, which often suffers from mass-transfer limitations, this work employs ultrasonic cavitation to generate localized high temperatures and pressures, accelerating oxidation kinetics and improving sulfur removal. The Fe–Co–Ni composite catalyst provides abundant redox-active sites that synergize with H₂O₂ to generate reactive oxygen species (ROS), while ultrasound ensures efficient dispersion of immiscible phases. This dual strategy not only reduces solvent consumption and reaction time but also achieves ultra-deep desulfurization of real diesel fuel. Furthermore, the systematic study of critical parameters (reaction time, temperature, catalyst dosage, oxidant ratio, and solvent extraction) offers a comprehensive understanding of process dynamics. To the best of our knowledge, the combined application of UAOD with Fe–Co–Ni composite catalysts has not been previously reported, making this work a pioneering contribution to sustainable clean fuel production.

## Experimental

### Materials

All of the reagents (Nickel nitrate Ni (NO_3_)_2_ 99%, Iron nitrate Fe (NO_3_)_3_ 99%, Cobalt nitrate Co (NO_3_)_2_ > 98%, Sodium hydroxide NaOH 95%) were acquired from Sigma Company, UK and all the reagents were used without further purification. On the other hand, the diesel fuel feedstock was obtained from Cairo oil refinery, Cairo- Egypt. The main characteristics of diesel fuel as feedstock in this study are indicated in Table 1

**Table 1:** Characterizations of gas oil.

Characterization	Diesel fuel	ASTM reference
Yield, Wt. %	100	–
Refractive index, 20 °C	1.3662	D-1218
Density, 20 °C, g/cm^3^	0.7110	D-1298
Aniline point, ^o^C	72	D-611
Sulfur content, ppm	21,700	D-4294

### Synthesis of Ni–Co–Fe mixed oxides composites

In order to prepare the proposed composites, 0.2 M solution of each metal salt was prepared via dissolving the corresponding amount of each salt in de-ionized water. The three solutions were then mixed together and heated up to 70 °C to increase the levels of salts solubility. Subsequently, 0.1 M sodium hydroxide (NaOH) solution was added drop-wise to the mixture at different time intervals in order to precipitate the metals in their hydroxide forms (pH of the mixture was adjusted at 11–12). Specifically, different reaction times (0.5, 1, 2, 4, and 8 h) were employed. The obtained precipitates were next collected via filtration and were washed by de-ionized water several times. A drying stage was then carried out for all samples inside an electric oven (set at 100 °C) and it was lasted for 24 h. The dried samples were, at the end, forwarded to a calcination stage at 500 °C for a time of 4 h^28,29^. The obtained mixed oxides were denoted according to the reaction time as follow ( 0.5 HOUR » T1, 1 HOUR » T2, 2 HOUR » T3, 4 HOUR » T4 and 8 HOUR » T5).

### Preparation of polystyrene-metal oxides core–shell

**Fig. 1:**
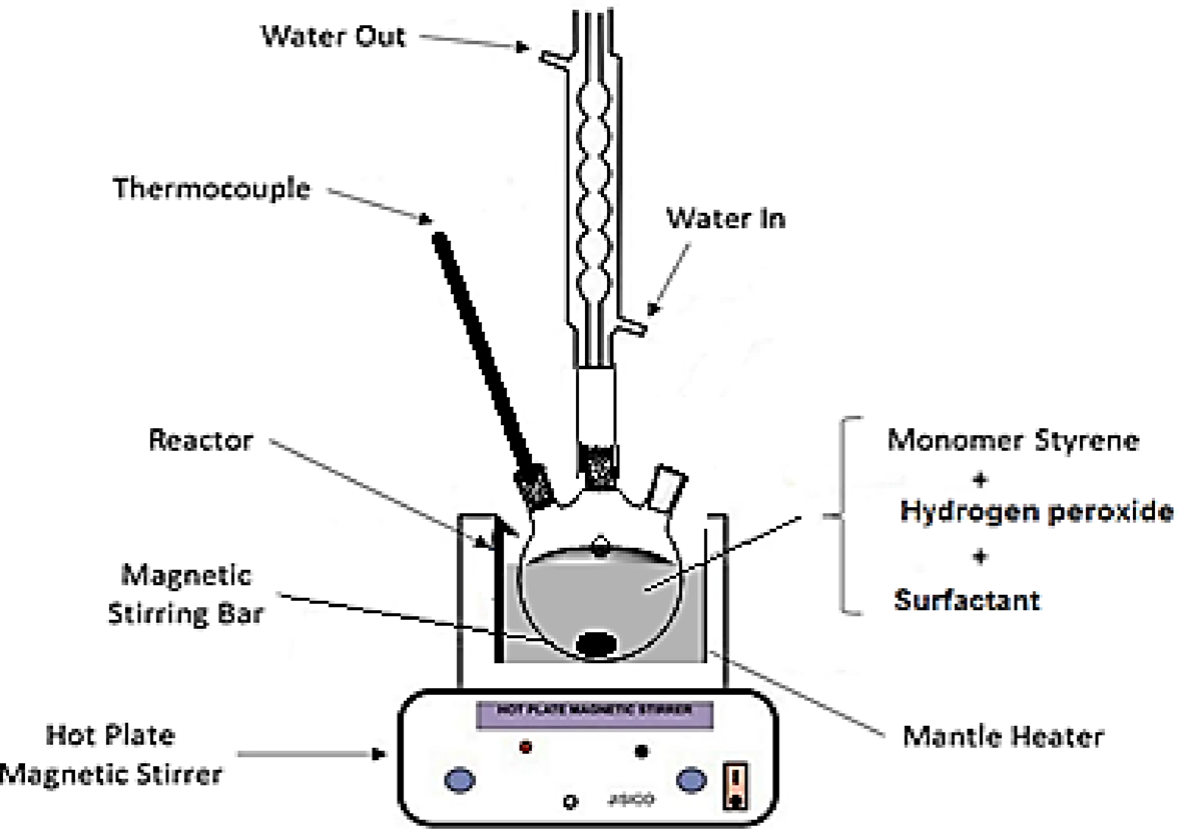
Experimental setup for the preparation of polystyrene-modified mixed oxide core–shell catalyst.

Polystyrene-metals oxides core–shell structure was synthesized using the High Internal Phase Emulsion (HIPE) polymerization technique. The synthesis procedures were launched by preparing two phases namely; aqueous and oil phase on separate basis. The aqueous phase contained double distilled water and H_2_O_2_ (as initiator) with respective ratios of 95:5% by weight. Also, this phase contained particles of Ni–Co–Fe composite, representing 20 Wt. % of its total weight. These particles were suspended into the aqueous phase via sonication (waves power of 70 W and frequency equals 40 kHz) for 20 min. The second phase (oil phase) was prepared by well-mixing of styrene monomer (90%) with 10% anionic-alkyl sulfonates (surfactant) until homogeneous solution was observed. The polymerization process was then executed using the schematically presented setup in Fig. 1. At the first place, the aqueous phase was inserted into the displayed glass reactor (three-necked round bottom flask), Scheme I, which is placed inside a temperature controlled heating mantle. The glass reactor was then heated under vigorous string (400 rpm) using magnetic stirrer until a reaction temperature of 60 °C was reached. Promptly, the oil phase was added to the reactor where an emulsion was immediately formed. The emulsion was subsequently kept to stir, at the prior-stated temperature, for 2 h under reflux to complete the polymerization reaction. By end of reaction time, nearly 90% of the polymer could be produced as dispensed into the remains of aqueous phase. The whole solution was then transferred into 500 mL beaker which was placed overnight inside an electric furnace at 60–65 °C. This step was meant in order to finalize the polymerization process and to produce a sample of dry powder (composite polymer). During the preparation stage, the concentrations of oil to aqueous phases in the composition of emulsion were set as 30:70 Wt. %.

### Characterization

The textural properties of the prepared samples were evaluated by N₂ adsorption–desorption isotherms at –196 °C using a NOVA 3200e surface area and pore size analyzer (Quantachrome Instruments, USA). The specific surface areas (S_BET_ ​) were calculated from the adsorption branch of the isotherms by applying the BET equation using the multi-point method at relative pressure P/P_0_​ = 0.05–0.3. Pore size distribution was determined from the desorption branch using the Barrett–Joyner–Halenda (BJH) method. The structural characteristics of the synthesized composites were examined using Fourier-transform infrared spectroscopy (FT-IR) on an AT1 Mattson Genesis Series spectrophotometer (Thermo Electron Corporation, USA). The morphological features were observed using scanning electron microscopy (SEM) with an XL30 ESEM-FEG environmental scanning electron microscope (Philips, Japan), which provides high-resolution imaging under variable pressure (up to 10 Torr) and elevated temperatures (up to 1000 °C). The crystalline structures of the samples were analyzed using X-ray diffraction (XRD) performed on a PANalytical X’Pert PRO diffractometer (PANalytical, Netherlands) equipped with Cu Kα radiation (λ = 1.5406 Å). The surface charge (zeta potential) and particle size distribution of the composites were determined using a Zetasizer Nano ZS90 (Malvern Instruments, UK). The magnetic properties of the samples were measured using a vibrating sample magnetometer (VSM), Lake Shore 7404 (Lake Shore Cryotronics Inc., USA) at room temperature under an applied magnetic field up to ± 15 kOe. The saturation magnetization (Ms) values were obtained from the plateau region of the magnetization–field (M–H) curves, while the coercivity (Hc) values were determined from the field strength corresponding to zero magnetization on the hysteresis loops.

### Desulfurization experiments

As soon as the featured characteristics of the prepared Ni–Co–Fe composites were determined, they were utilized in the process of ultrasonic assisted oxidative desulfurization (UAOD) of a diesel fuel feedstock has a sulfur content of 21,700 ppm. The Ni–Co–Fe composites were first presented to the desulfurization at fixed operating conditions. Specifically, 10 g/ L, 60 °C, H_2_O_2_ to feed ratio of 1:1 and reaction time for 1 h were applied at the first place. Then, the sample which showed the highest desulfurization percentage was tested at different reaction times. Particularly, the effect of different reaction times (0.5, 1, 1.5, 2 and 2.5 h) on the desulfurization percentages was investigated. The acquired products were forwarded for analysis where the optimum time was determined. This time was then utilized in the next stage at which the effect of temperature change on the desulfurization process was investigated. Exactly, the catalytic UAOD process was performed at temperatures of 30, 45, 60 and 75 ºC whereas the catalyst dose was kept the same as the prior stage. By the completion of the latter stage, the most proper desulfurization temperature was picked. Both optimal temperature and time were next used to explore the impact of catalyst to feed dose (5, 7.5, 10 and 12.5 g/ L) on the sulfur removal process. Also, the effect of H_2_O_2_ (as an oxidizing agent) to feed ratio on sulfur removal was studied. The optimum operating conditions through all the previous stages were next utilized to perform the UAOD process while using the prepared polystyrene-modified mixed oxide core–shell catalyst. Ultimately, the influence of solvent extraction process, as a supplementary step, on the reduction of sulfur content in the produced diesel fuels is studied. Generally, the removals of sulfur compounds during all the experiments were identified using X-ray fluorescence spectrophotometer device model XR-ECO SC632, Germany.

## Results and discussion

The current research study investigates the treatment of diesel fuel feedstock in order to reach the highest possible desulfurization percentage. Such process could be counted of a high importance from environmental perspectives since the hazardous impacts of diesel fuels can be reduced. Different composites made of Ni, Fe and Co mixed oxides nanoparticles are introduced as efficient structures for the implementation of oxidative/ sono-catalytic desulfurization process.

### Characterization of Ni–Co–Fe composites

**Fig. 2:**
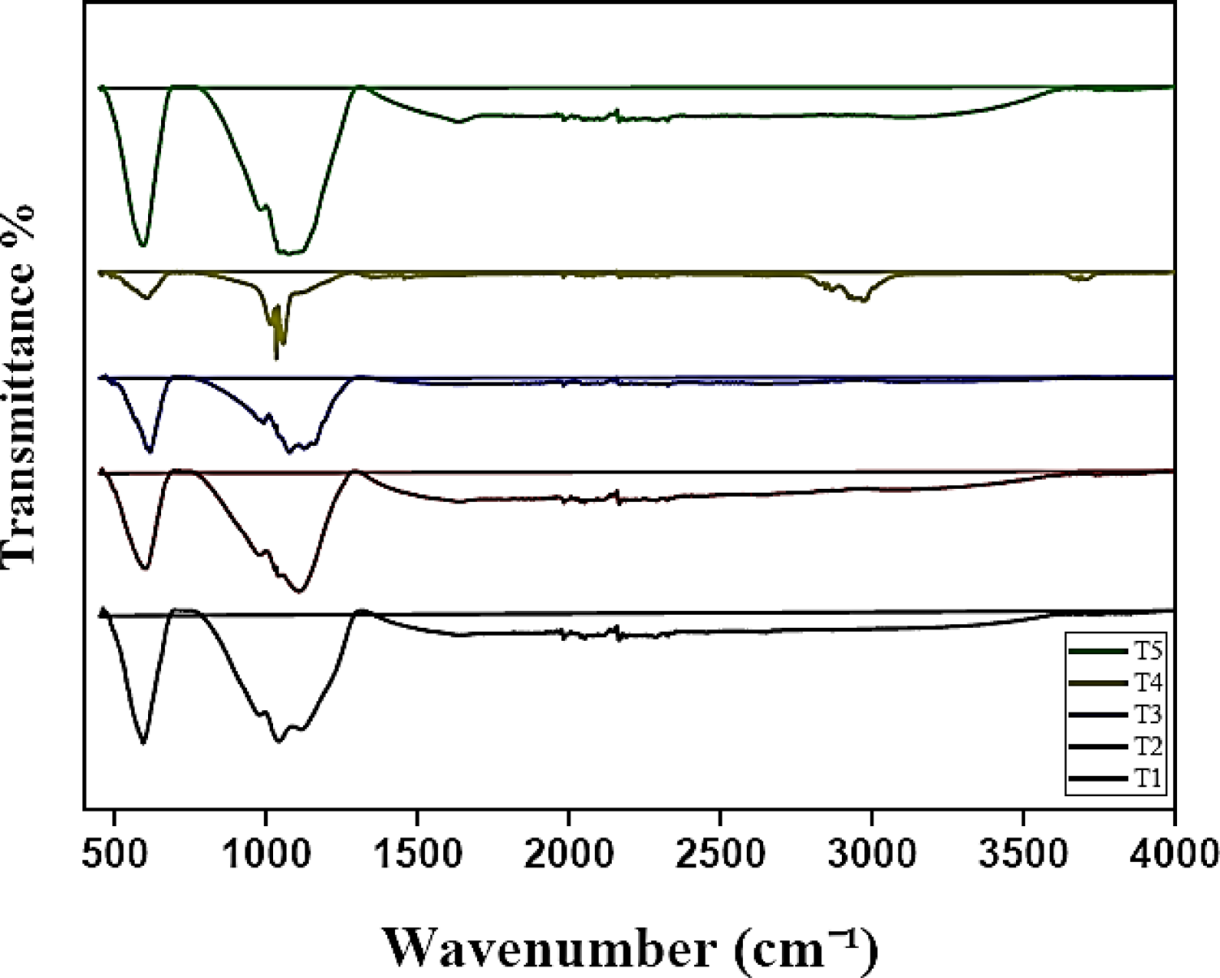
FTIR spectra of the prepared Ni–Co–Fe composites (T1-T5).

The FTIR spectra of the synthesized Ni–Co–Fe mixed-metal oxide composites (T1–T5) revealed critical insights into their structural and bonding characteristics (Fig. 2). Absorption bands in the 450–600 cm⁻^1^ range were assigned to Fe^3^⁺–O stretching vibrations in hematite (α-Fe_2_O_3_) )JCPDS No. 33–0664(, consistent with studies on iron oxide vibrational modes^30^. On the other side, the 550–700 cm⁻^1^ region indicated Co^2^⁺/Co^3^⁺–O vibrations from spinel-type Co_2_O_3_) JCPDS No. 20–0435(, aligning with literature on cobalt oxide phases^31^. Ni^2^⁺–O bonds in cubic NiO) JCPDS No. 47–1049 (were identified near 400–500 cm⁻^1^, corroborating infrared studies of nickel oxide nanostructures^29,30^. The minor shifts in these bands across the composites (e.g., Fe–O at 500–612 cm⁻^1^ in T1 vs. T5) suggest lattice distortions due to ionic radius mismatches (Fe^3^⁺: 0.64 Å, Co^2^⁺: 0.74 Å, Ni^2^⁺: 0.69 Å), leading to strain and altered bond lengths which are generally observed in mixed-metal oxide systems^32,33^. Broadening of peaks in T5 (8 h synthesis) versus T1 (0.5 h) reflects increased agglomeration and reduced crystallinty, a phenomenon linked to prolonged synthesis times promoting magnetic interactions and particle coalescence^34^. Additionally, weak bands near 3400 cm⁻^1^ and 1600 cm⁻^1^, attributed to adsorbed moisture or residual hydroxyl groups^35,36^, highlight incomplete calcination—a common artifact in wet-chemical syntheses.

**Fig. 3:**
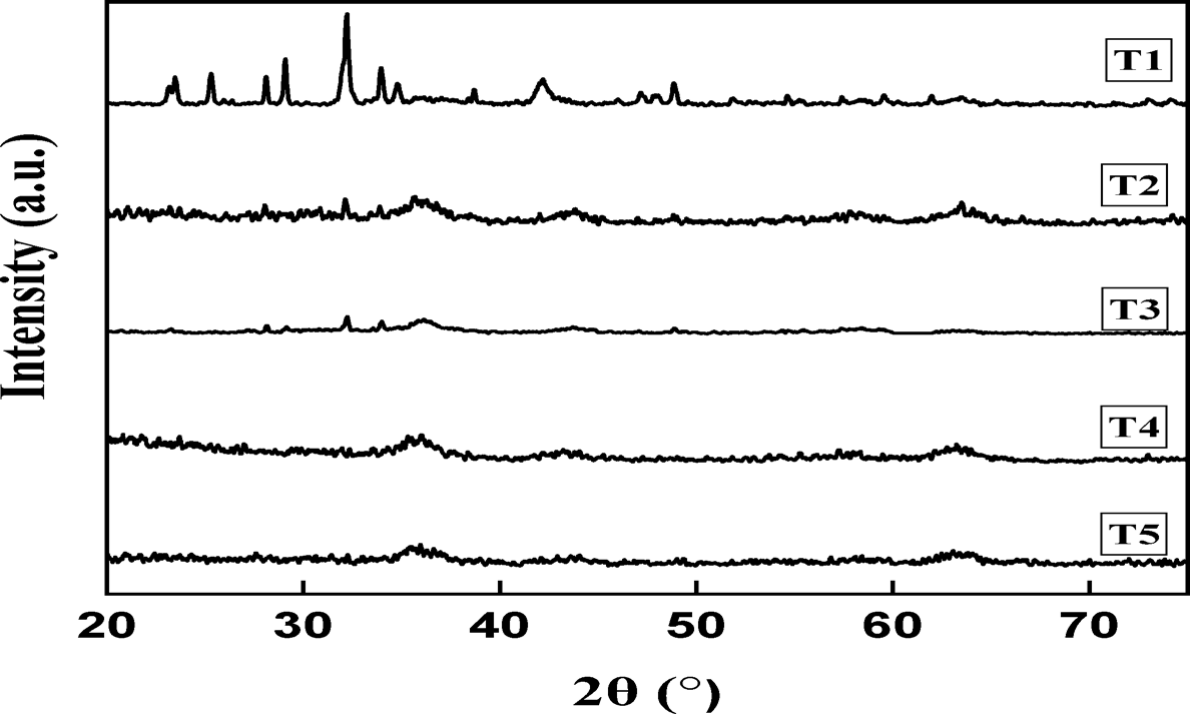
XRD charts of the prepared Ni–Co–Fe composites (T1-T5).

The X-ray diffraction (XRD) patterns of the Ni–Co–Fe mixed-metal oxide composites (T1–T5) provide critical insights into their crystallinity (Fig. 3), phase composition, and structural evaluation. The diffraction profiles reveal that the catalysts exhibit predominantly amorphous character, as evidenced by the broad and weak diffraction peaks, with only minor features corresponding to poorly crystalline phases. This amorphous nature is advantageous for catalytic applications, since it increases surface area, introduces structural defects, and enhances the dispersion of active metal sites. This in turn contributes to improved accessibility and reactivity during oxidative desulfurization processes. Key peaks observed at 2θ = 35° and 44° correspond to the (311) plane of spinel Co₂O₃ (JCPDS No. 42–1467)^37^ and the (200) plane of cubic NiO (JCPDS No. 47–1049)^37^, respectively. The detected signal at 2θ ≈ 20° corresponds to the (104) plane of hematite (α-Fe₂O₃), revealing amorphous nature in terms of observing broad hump-like peak. Hematite reflections (α-Fe₂O₃) are instead confirmed from the diffraction pattern near 2θ ≈ 33° ((104), JCPDS No. 33–0664)^38^. In addition, there is distinct peak at 2θ ≈ 62.6**°** in all catalysts which is assigned to the (214) plane of hematite (α-Fe₂O₃), (JCPDS No. 33–0664) overlapping with the (440) plane of CoFe₂O₄ spinel (JCPDS No. 22–1086) and minor contributions from the (220) plane of cubic NiO (JCPDS No. 47–1049**)**. The coexistence of these XRD signals confirms the formation of a mixed-phase composite without dominant single-phase segregation, aligning with reports on ternary metal oxide systems^39^. Observation of additional peaks at 2θ = 33°, 48°, and 52° further validate the presence of secondary phases, such as gamma-hematite (γ-Fe2O3) (JCPDS No. 39–1346) and CoFe2O4 spinel structures (JCPDS No. 22–1086), which are common in Fe–Co–Ni oxide composites^37,40^.

The broadening of peaks from T1 (0.5 h synthesis) to T5 (8 h synthesis) indicates increased lattice strain and reduced crystallite size, as described by the Scherrer equation D = Kλ / (B cosθ), which reflects the size of individual crystalline domains within particles. Using this equation, the crystallite sizes were calculated from the FWHM of the (311) Co₂O₃ peak, showing a significant broadening in T5 and a corresponding decrease in crystallite size from ~ 25 nm (T1) to ~ 12 nm (T5) due to prolonged synthesis-induced agglomeration^41^^.^ T5 shows dominant CoFe_2_O_4_ spinel peaks (2θ = 35°, 48°, 52°), evidenced by intensified (311) and (400) reflections and suppressed α-Fe_2_O_3_/NiO signals. The detected average crystal sizes correlates with BET data, where T1’s higher surface area (34.22 m^2^/g) corresponds to smaller well-dispersed crystallites, while T5’s lower surface area (11.27 m^2^/g) reflects agglomerated particles^42^. On another side, the noted structural shift from mixed oxides (T1) to CoFe_2_O_4_-rich phases (T5) underscores a trade-off: short synthesis times favor catalytic activity in terms of obtaining increased surface area.

**Fig. 4:**
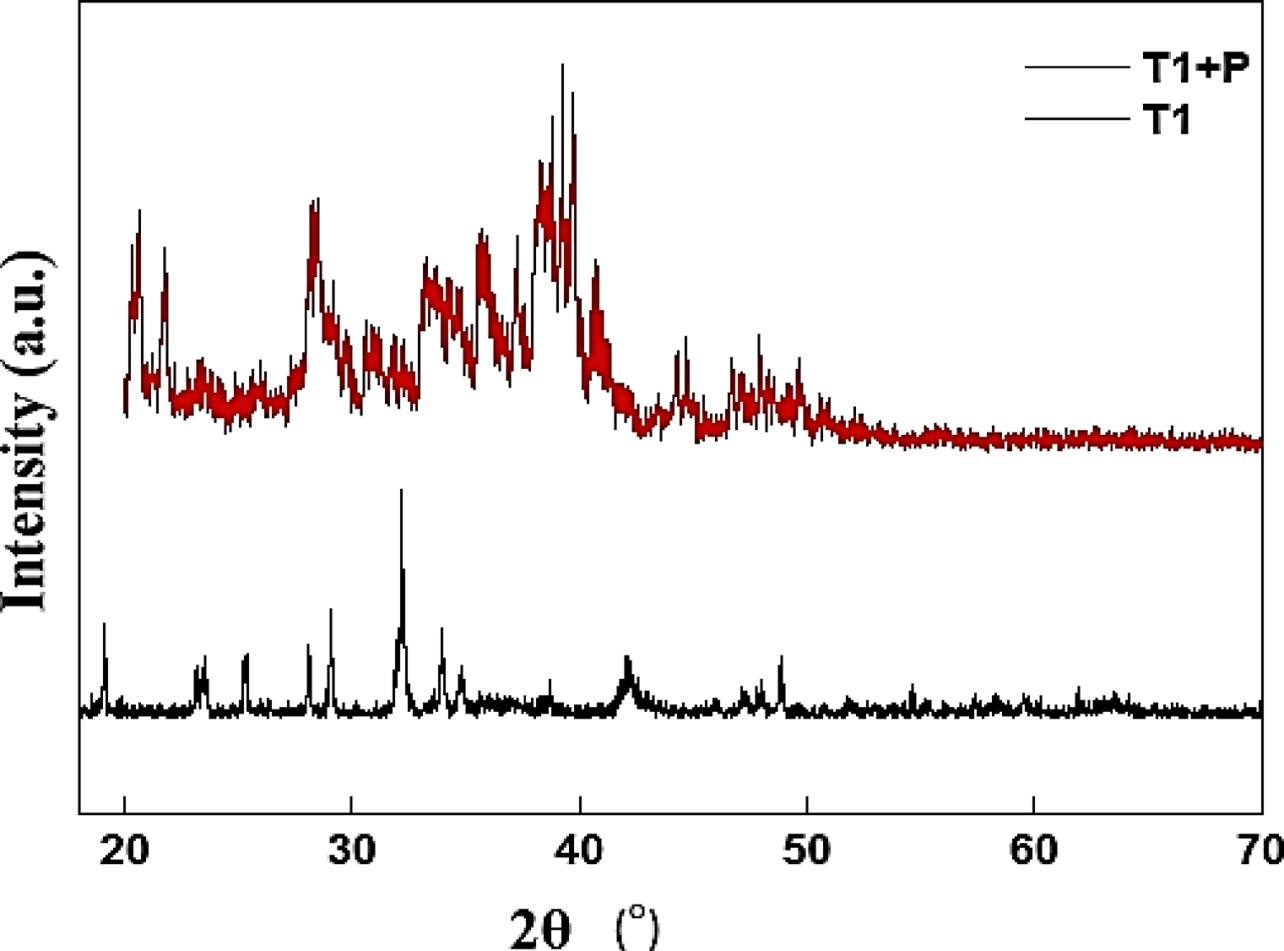
XRD charts of sample T1 and sample T1 after modification with the polystyrene polymer (TS!).

The XRD analysis of the Ni–Co–Fe composites (Fig. 4) catalyst shows sharp and intense peaks corresponding to crystalline phases of NiO, Co₂O₃, and Fe₂O₃, with distinct reflections such as (104) for α-Fe₂O₃, (311) for Co₂O₃, and (200) for NiO. These results confirm that Ni–Co–Fe composite (T1) possesses a well-defined mixed-metal oxide structure with high crystallinity. In contrast, the polystyrene-modified T1 (core–shell) exhibits reduced peak intensities for these crystalline oxides, which is attributed to the amorphous nature of polymer layer. A broad hump in 2θ range of 20–30°, characteristic of amorphous polymers, further validates the presence of the polystyrene shell. This apparent reduction in crystallinity correlates with SEM findings of polymer-induced surface amorphization, while the persistence of the main oxide peaks confirms that the crystalline core remains intact. Thus, the polystyrene coating modifies the surface characteristics without degrading the underlying oxide framework, enabling synergistic catalytic behavior^43,44^.

**Fig. 5:**
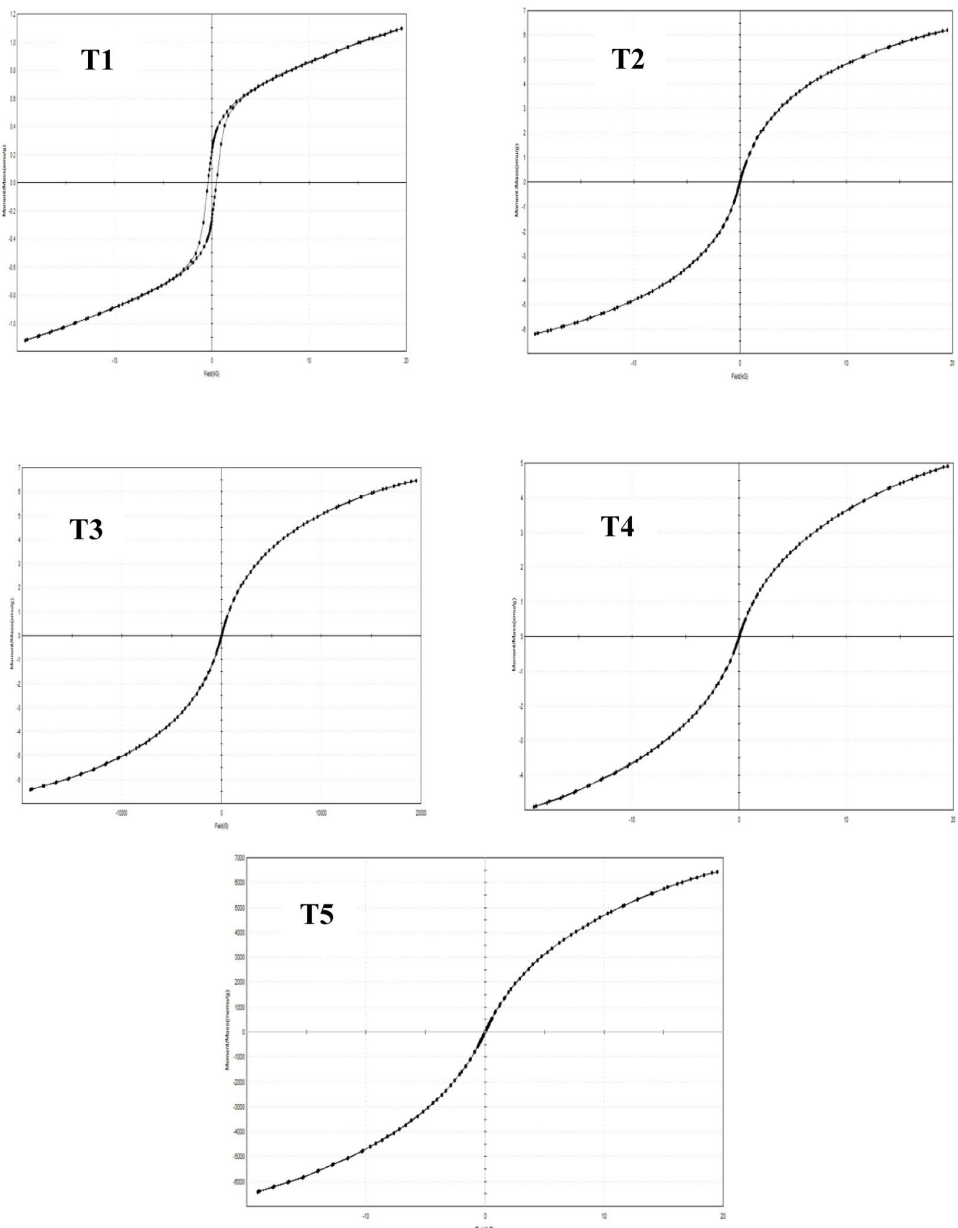
Magnetic behaviors of the prepared Ni–Co–Fe composites (T1-T5).

The Ni–Co–Fe composites (T1–T5) exhibit progressive evolution in structural, magnetic, and catalytic properties, reflecting the interplay of synthesis duration and particle dynamics. T1 displayed the highest surface area (34.22 m^2^/g) and smallest crystallite size (~ 25 nm), attributed to rapid nucleation which limited particle growth^45^**.** This nanostructure may enhance the catalytic activity due to abundant exposed active sites and mesopores (pore radius ~ 10 nm), which facilitated sulfur compound adsorption and oxidation. However, its lower saturation magnetization (Ms = 45 emu/g) and coercivity (Hc = 220 Oe) indicated weaker magnetic recoverability (Fig. 5), as surface spin disorder and smaller domain sizes suppressed interparticle interactions. The T2 (1 h) and T3 (2 h) marked transitional phases^46,47^. T2’s surface area (25.67 m^2^/g) and pore volume (0.0619 cm^3^/g) decreased moderately compared to T1, reflecting initial agglomeration, while crystallite size increased to ~ 20 nm. XRD peak broadening (e.g., Fe_2_O_3_ at 2θ = 35°) suggested lattice strain from partial Co/Ni incorporation into the Fe-oxide matrix, a phenomenon observed in ternary systems with ionic radius mismatches. Magnetic properties improved slightly, with Ms rising to 52 emu/g (T2) and 58 emu/g (T3), as longer synthesis times promoted crystallite growth (~ 22 nm for T3) and stronger exchange interactions. T4 (4 h) exhibited pronounced agglomeration, with surface area dropping to 14.33 m^2^/g and pore radius widening to 19 nm, indicative of interparticle sintering. XRD peaks further broadened, revealing increased amorphous content and strain from competing magnetic interactions (e.g., Fe^3^⁺–O–Co^2^⁺ linkages). Ms was surged to 65 emu/g (T4) due to increased crystallites (~ 35 nm), forming multidomain structures with reduced surface-to-volume ratios. The enhanced magnetism (Hc = 380 Oe) of T4 could improve its recyclability after processing. T5 (8 h), prioritized magnetic properties, showing high Ms (72 emu/g) and Hc (450 Oe) enabled efficient magnetic separation which is critical point for its use in industrial scales. The dominance of spinel phases (e.g., CoFe_2_O_3_) in T5, confirmed by XRD and FTIR, underpinned its robust ferromagnetism since that large domains reduced spin canting and surface anisotropy^47,48^.

**Fig. 6:**
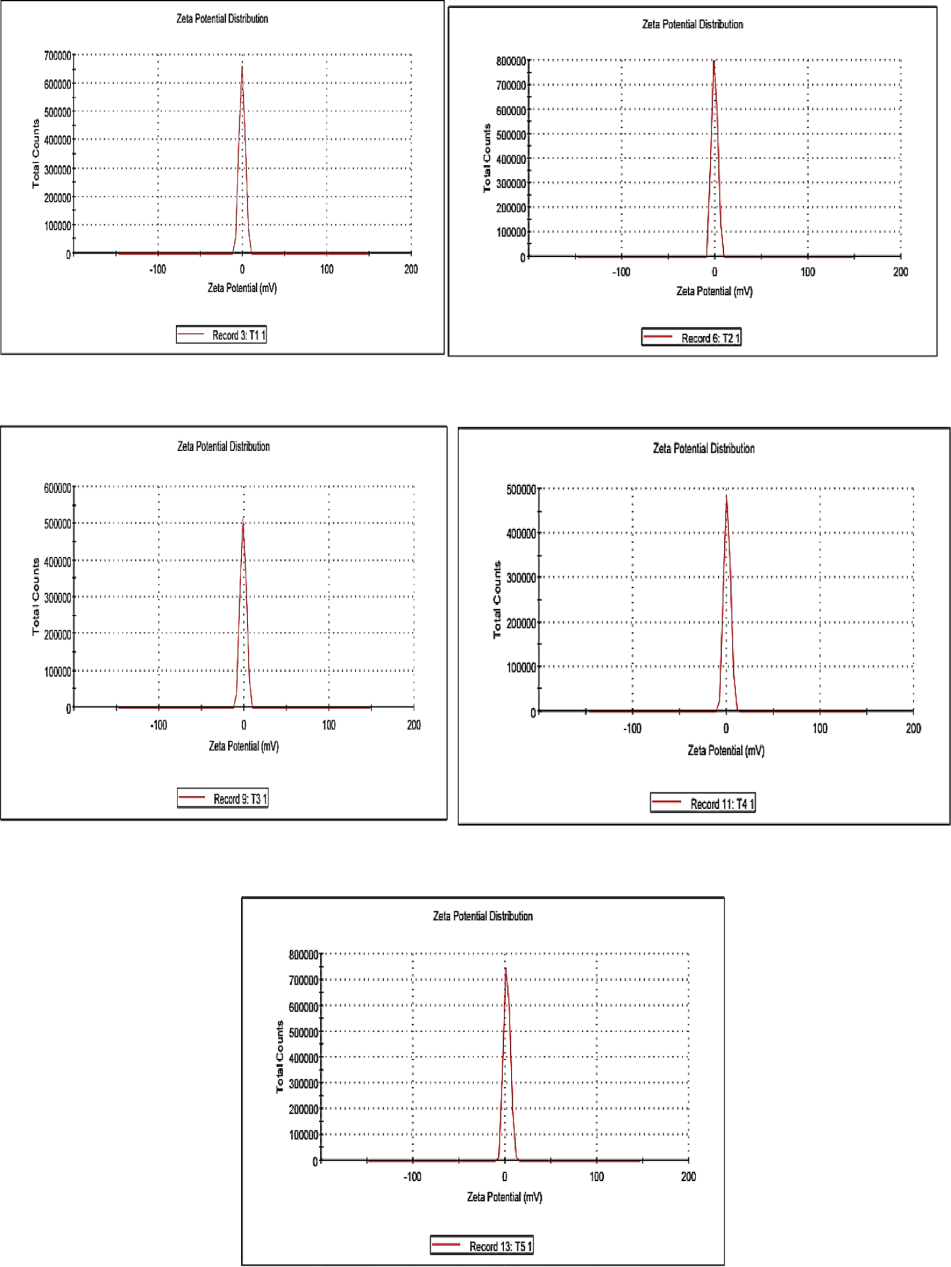
Zeta potential curves of the prepared Ni–Co–Fe composites (T1-T5).

The zeta potential analysis of the Ni–Co–Fe composites reveals near-neutral surface charges (− 1 to + 0.3 mV) as shown in Fig. 6, reflecting a balance between oxygen-rich moieties and metal cation dominance, governed by synthesis time, particle agglomeration, and surface chemistry. Catalysts synthesized over shorter durations (0.5–1 h) exhibit marginally negative potentials (− 1 to − 0.3 mV) due to residual hydroxyl groups and oxygen-terminated surfaces, which weakly interact with polar sulfur species such as sulfones. These systems, characterized by smaller particle sizes (~ 100–150 nm) , leading to high surface areas which can in turn maximize sulfur removal but suffer. However, they suffer from limited magnetic saturation (45–52 emu/g), complicating their recyclability^49,50^. As synthesis time extends (2–4 h), progressive agglomeration (~ 180–220 nm sized particles) and calcination reduce hydroxyl density, shifting zeta potentials toward neutrality (− 0.5 to 0 mV). This transition enhances magnetic properties (58–65 emu/g saturation, 320–380 Oe coercivity) through crystallite growth (~ 22–35 nm) and spinel phase formation. Hence, sulfur removal may decline due to reduced surface accessibility. Catalysts with the longest synthesis duration (8 h) exhibit near-neutral zeta potentials (+ 0.3 mV) from oxygen-deficient, cation-dominated surfaces, alongside significant particle coalescence (~ 250 nm) and collapsed pore structures (20 nm radii). These systems achieve robust magnetism (72 emu/g saturation, 450 Oe, coercivity) but may minimize catalytic sulfur removal efficiency, underscoring the inverse relationship between magnetic recoverability and reactivity. Importantly, since all Ni–Co–Fe composites exhibit near-neutral zeta potential, electrostatic interactions play only a minor role in capturing sulfur compounds. Thus, the dominant mechanisms governing sulfur removal are non-electrostatic in nature, pore confinement and π–π interactions rather than electrostatic attraction, highlighting the nuanced interplay between surface chemistry and adsorption pathways. These findings emphasize the importance of tailoring synthesis parameters and functionalization strategies to optimize catalytic activity, selectivity, and recyclability for sustainable fuel purification^51,52^.

**Fig. 7:**
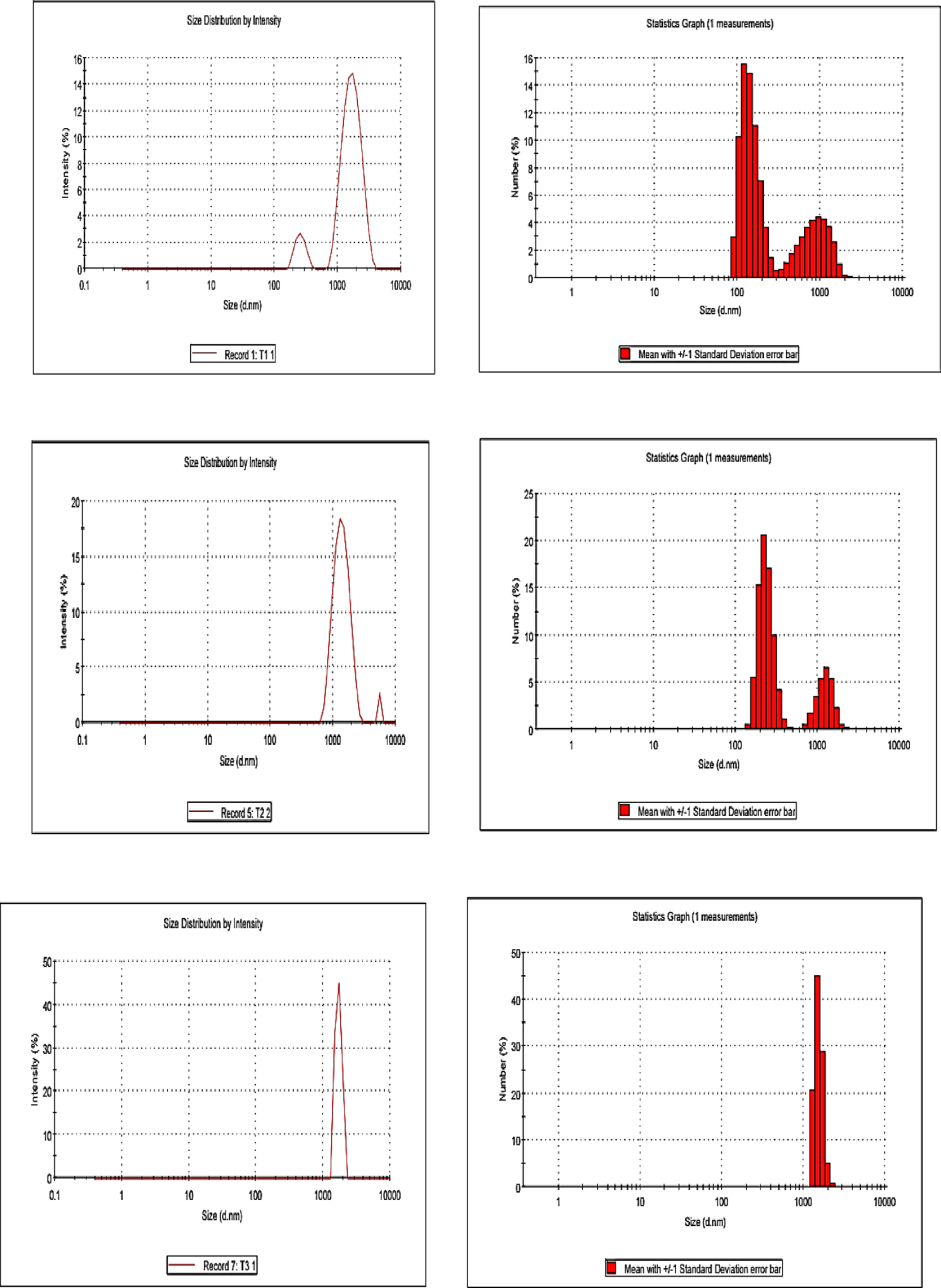

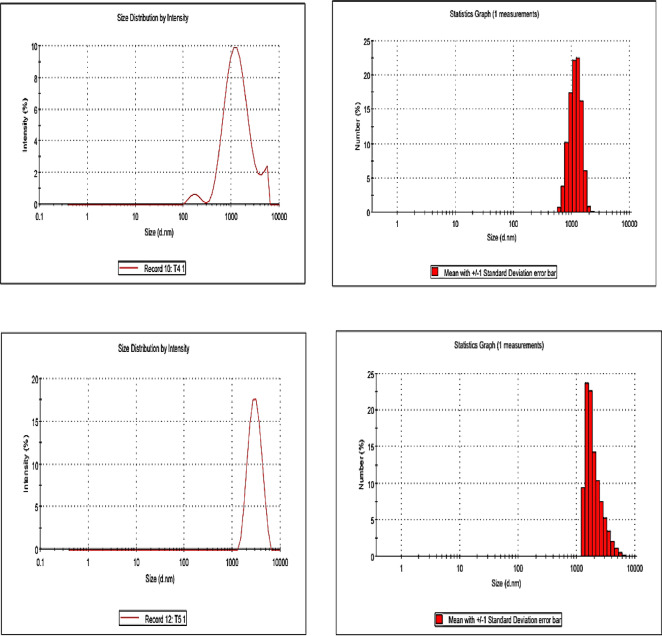
particle size distribution of prepared Ni–Co–Fe composites (T1-T5).

The presented particle size distribution analysis (as conducted via DLS) in Fig. 7 reveals a systematic evolution in particle dimensions and agglomeration behavior across the Ni–Co–Fe composites (T1–T5) as synthesis time increases from 0.5 to 8 h. T1 (synthesized at 0.5 h), exhibits the narrowest particle size distribution (90–110 nm) with a low polydispersity index (~ 0.15), indicating uniform nucleation and suppressed growth. This homogeneity is attributed to rapid precipitation kinetics, which limit interparticle interactions and agglomeration. The smaller particle size also reduces magnetic dipole–dipole forces, stabilizing dispersion and minimizing coalescence^53,54^.

As synthesis time extends (T2: 1 h and T3: 2 h), particle size distributions broaden slightly, averaging 130–170 nm (T2) and 170–200 nm (T3), with polydispersity indices increasing to ~ 0.20–0.25. This shift reflects the onset of Ostwald ripening, where smaller crystallites dissolve and redeposit onto larger ones. In parallel, weak magnetic interactions begin to drive partial agglomeration. These processes indicate a transition from kinetically controlled to thermodynamically favored growth, consistent with extended reaction times.

At longer synthesis durations (T4: 4 h and T5: 8 h), the particle size distribution becomes significantly broader (200–250 nm for T4; 230–280 nm for T5), with high polydispersity indices (~ 0.30–0.35). This behavior highlights uncontrolled agglomeration dominated by magnetic dipole coupling between Fe^3^⁺–Co^2^⁺ and Fe^3^⁺–Ni^2^⁺ ions, as well as van der Waals attractions. Moreover, near-neutral zeta potentials observed for T4 and T5 reduce electrostatic stabilization, further facilitating aggregation. The combined effect of magnetic interactions, surface energy minimization, and sintering explains the formation of dense, irregular aggregates under prolonged synthesis conditions^55–57^. In a summary, the large particle sizes and high polydispersity explain the observed aggregation and reduced surface accessibility, potentially leading to low catalytic activity in sulfur removal. Thus, the synergy between particle size and surface charge distribution (zeta potential) provides a comprehensive understanding of how synthesis duration dictates the balance between catalytic activity, stability, and recyclability.

The presented Brunauer–Emmett–Teller (BET) surface area analysis of Ni–Co–Fe composites (T1–T5) in Table 2 reveals a progressive decline in surface area and pore accessibility as synthesis time increases from 0.5 to 8 h. T1 (0.5 h), synthesized under the shortest duration, exhibits the highest surface area (34.22 m^2^/g) and smallest average pore radius (10 nm), indicative of a mesoporous structure with well-dispersed nanoparticles^58^. This high surface area arises from rapid nucleation which suppresses particle growth, creating a network of narrow pores ideal for trapping smaller sulfur compounds (e.g., thiophenes) via confinement effects. The moderate pore volume (0.0834 cm^3^/g) further supports efficient mass transfer of reactants to active sites, enhancing its potential for superior sulfur removal efficiency. T2 (1 h) and T3 (2 h) show intermediate surface areas (25.67–17.65 m^2^/g) and pore radii (16–18 nm), reflecting incremental particle agglomeration as synthesis time extends. The broader pore size distribution in these catalysts suggests partial sintering, where smaller pores merge into larger ones, reducing surface site density but can accommodate bulkier sulfur molecules (e.g., dibenzothiophene). The decline in pore volume (0.0619–0.0464 cm^3^/g) may lead to a reduced sulfur removal activity, as a few numbers of active sites can be accessible for adsorption and oxidation. T4 (4 h) and T5 (8 h) exhibit the lowest surface areas (14.33–11.27 m^2^/g) and largest pore radii (19–20 nm), characteristic of extensive agglomeration and interparticle sintering^59^. Prolonged synthesis durations do not promote crystallite growth but instead drive agglomeration of smaller strained crystallites into larger secondary aggregates. This aggregation enhances magnetic interactions and progressively collapses the mesoporous framework into broader pore domains (> 20 nm). Therefore, surface accessibility for sulfur compounds can be strongly reduced. The diminished pore volumes (0.0398–0.0322 cm^3^/g) in these samples restrict reactant diffusion to active sites, resulting in a potentially low sulfur removal efficiencies^60,61^.

**Table 2:** Surface characteristic of the prepared composites (T1-T5) at different processing times.

Sample code	S_BET_ (m^2^/ g)	V_P_ cm^3^/ g)	r_p_ (nm)
T1	34.22	0.0834	10
T2	25.67	0.0619	18
T3	17.65	0.0464	16
T4	14.33	0.0398	19
T5	11.27	0.0322	20

**Fig. 8:**
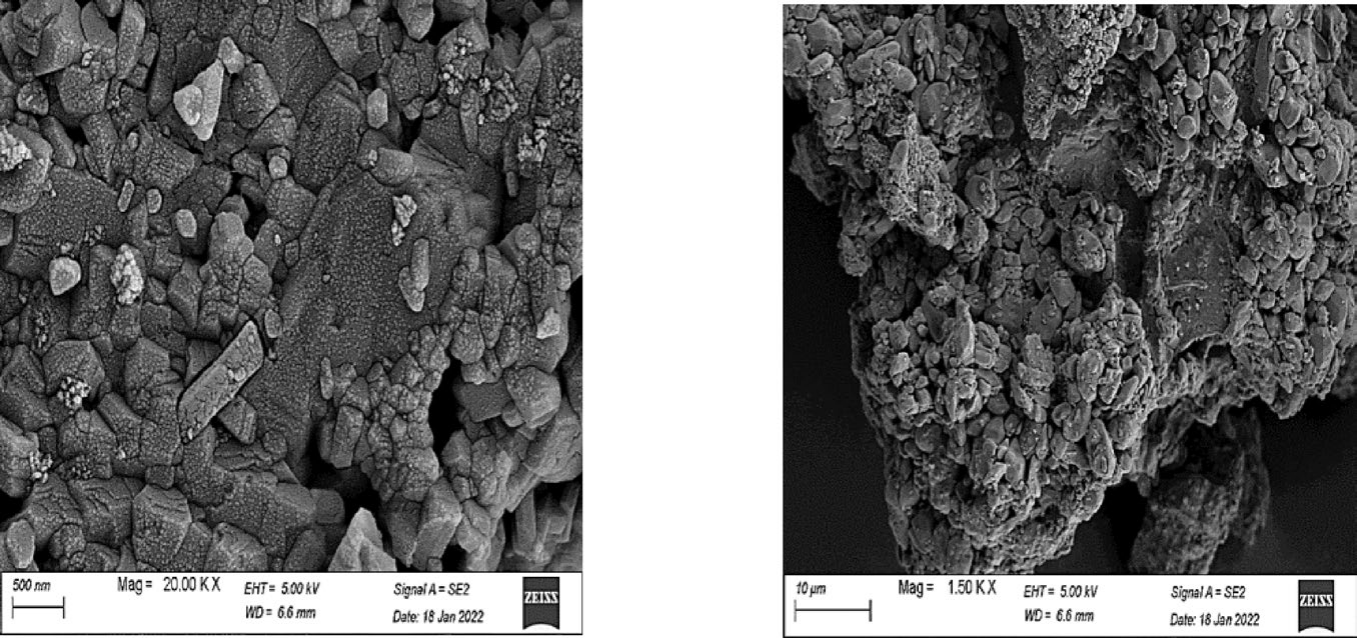
Surface morphology of sample T1.

**Fig. 9:**
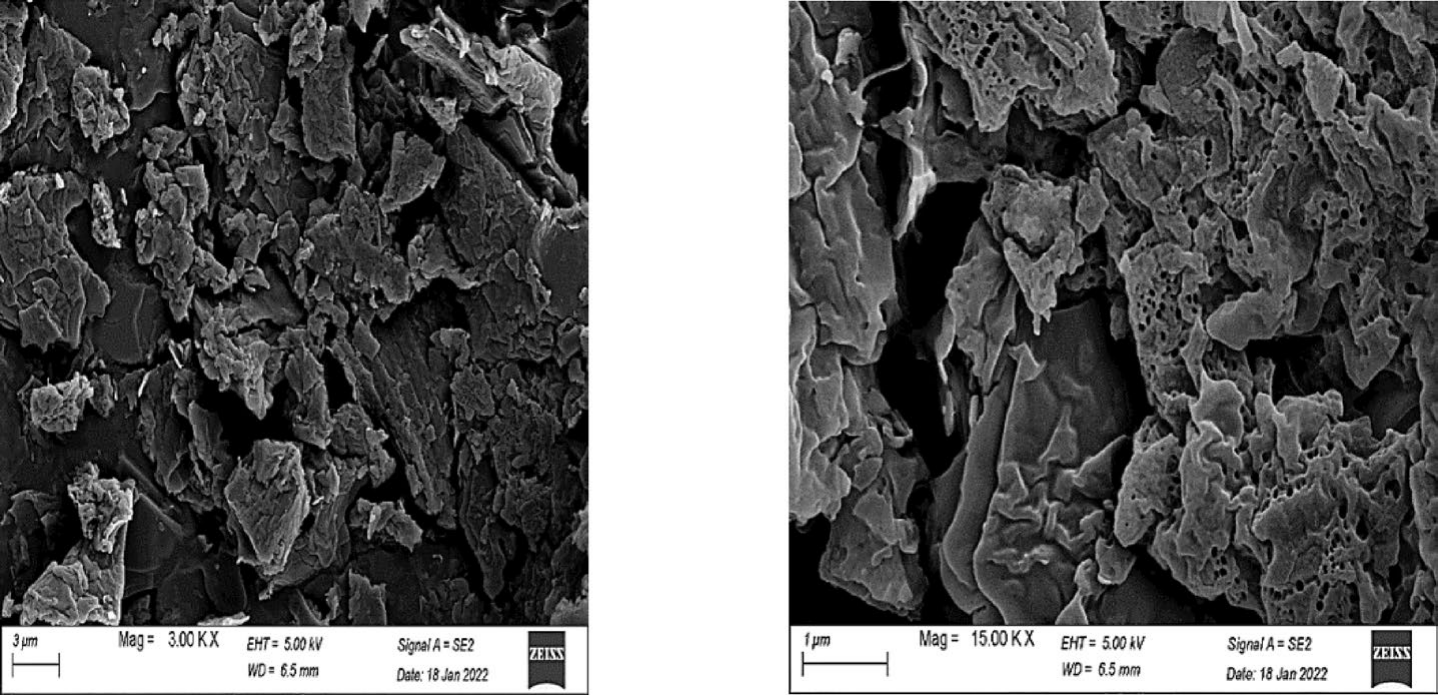
Morphology change of sample T1 after modification with the polystyrene polymer.

The displayed SEM analysis in the Figs. 8 and 9 are explicitly reported for T1 (unmodified) and T1 modified with polystyrene (polymer-mixed oxide core–shell structure). For the unmodified T1, SEM images reveal well-dispersed nanoparticles (~ 50–100 nm) with uniform morphology, displaying distinct tetragonal and pentagonal geometries indicative of crystallographic growth during rapid precipitation. This morphology aligns with its high surface area and narrow pore distribution, which may facilitate sulfur adsorption via pore confinement and surface interactions. In contrast, the polystyrene-modified T1 exhibits a core–shell structure, where the metal oxide core is encapsulated by a rough, irregular polystyrene layer. The polymer shell introduces hierarchical porosity (macro-mesopores) via HIPE tenplating, enhancing diffusion of sulfur compounds during the removal process while retaining the core’s mesoporosity. The exhibited SEM images for the core–shell structure (modified T1) confirms the loss of crystallinity^62,63^. However, its architecture may result in obtaining high sulfur removal through synergistic mechanisms: pore confinement in the core and π-π interactions (polystyrene-aromatic sulfur compounds) in the shell. . On the other hand, a larger number of pores could be detected in the modified T1 composite in comparison to the blank metals oxides. The increase in number of pores (as detected in SEM images) can be referred to the method of polymer preparation, high internal phase emulsion polymerization^64^.The SEM data underscores the importance of controlling synthesis time (T1) and surface engineering (core–shell) to optimize morphology for high catalytic efficiency without agglomeration.

### Process of diesel fuel desulfurization

#### Effect of composite type

**Fig. 10:**
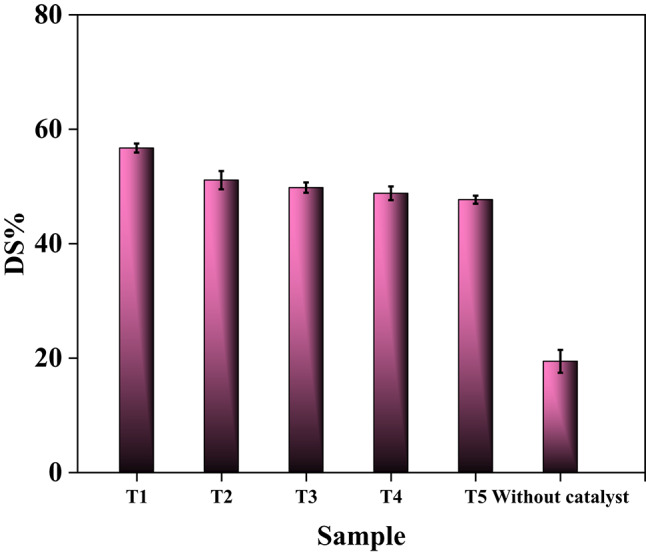
Effect of composite type on the percentage of sulfur removal (at T = 30ºC, t = 1h, dose = 10g/L, H_2_O_2_/Feed = 1:1).

The work systematically evaluates the impact of composite type (T1–T5) and structural modifications on sulfur removal efficiency during UAOD of diesel fuel. As shown in (Fig. 10 and Table S1) the sulfur removal efficiency in the absence of the catalyst was only 19.5%. T1, synthesized for the shortest duration (0.5 h), achieves the highest sulfur removal efficiency (56.7%) due to its optimal structural properties: the highest surface area (34.22 m^2^/g), smallest particle size (~ 100 nm), and narrow mesopores (10 nm radius). These features could effectively maximize sulfur compound adsorption via pore confinement and surface interactions (e.g., hydrogen bonding with residual hydroxyl groups) (Fig. 9).

As synthesis time increases (T2: 1 h; T3: 2 h; T4: 4 h; T5: 8 h), sulfur removal efficiency progressively declines (52 → 50 → 47.5 → 46%). This trend correlates with particle agglomeration, reduced surface area (25.67 → 11.27 m^2^/g), and pore widening (10 → 20 nm), which limit active site accessibility. While an increase in pore size generally facilitates reactant diffusion and accessibility to catalytic active sites, an excessive pore enlargement in the introduced catalytic systems led to a reduction in the density of active surface sites and weaker confinement effects. This structural change reduces the effective interaction between sulfur compounds and catalytic centers, thereby lowering the oxidation efficiency. Agglomeration in T4–T5 buries hydroxyl groups and oxygen moieties, shifting adsorption mechanisms to weaker metal-sulfur coordination rather than pore confinement or electrostatic interactions^65,66^. Despite of their reduced magnetization (38 emu/g vs. T1’s 45 emu/g), the design of these two composites prioritizes catalytic efficiency over magnetic recovery, demonstrating the utility of surface engineering^67^.

#### Effect of oxidizing agent amount on sulfur compounds removal

**Fig. 11:**
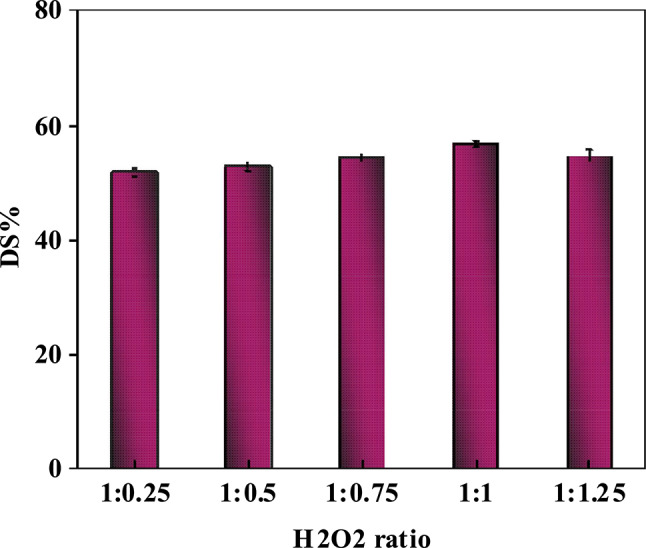
Influence of H_2_O_2_ contents on the percentage of sulfur removal (at T = 30ºC, t = 1h, dose = 10g/L).

The study establishes that sulfur removal efficiency in the ultrasound-based catalytic oxidative desulfurization is critically dependent on the H_2_O_2_-to-feed ratio. As seen in Fig. 11 and Table S2, 1:1 (H₂O₂-to-feed ratio) resulted in the highest sulfur removal efficiency (56.7%). It should be noted that the oxidant-to-feed ratio reported in this study refers to the volume ratio of H₂O₂ solution to diesel feed, rather than the stoichiometric oxidant-to-sulfur molar ratio. Theoretically, two moles of H₂O₂ are required to completely oxidize one mole of sulfur compound to the corresponding sulfone, as represented in Eq. (1):

$$R-S+2{H}_{2}{O}_{2}\to RS{O}_{2}+2{H}_{2}O$$

At this ratio, the Ni–Co–Fe composite activates H_2_O₂ via metal redox cycles (Fe^3^⁺/Fe^2^⁺, Co^3^⁺/Co^2^⁺), generating reactive oxygen species (ROS) such as hydroxyl radicals (•OH) that selectively oxidize refractory sulfur compounds (e.g., dibenzothiophene) into polar sulfones. These oxidized compounds are subsequently removed by adsorption onto the composite structure or via a subsequent solvent extraction step^68^. Ratios higher than 1:1 could reduce the desulfurization efficiency due to excessive oxidant decomposition into O₂/H₂O, leading to emulsification-induced phase separation, and side reactions with hydrocarbons. On the other hand, ratios lower than 1:1 led to incomplete sulfur oxidation, minimizing the sulfur removal efficiency. Ultrasonic cavitation further enhanced the process by dispersing H₂O₂, intensifying ROS accessibility, and accelerating reaction kinetics through localized high-pressure/temperature microenvironments^69^. The provided insights ensure the necessity of precise stoichiometric control to balance oxidation efficiency and reaction stability for deep desulfurization under mild, non-photocatalytic conditions^65^.

#### Effect of operating time on the desulfurization level

**Fig. 12:**
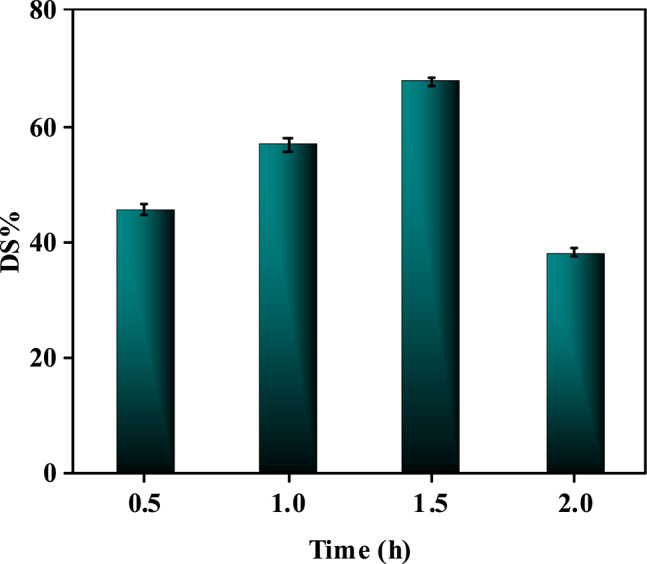
Impact of operating time change on the percentage of sulfur removal (at T = 30ºC, H_2_O_2_ to feed ratio of 1:1 and dose = 10g/L).

The operating time significantly influences sulfur removal efficiency (Fig. 12 and Table S3), with a distinct optimum window observed during ultrasound-based catalytic oxidative desulfurization. Initially, sulfur removal increases with prolonged reaction time due to enhanced interaction between the catalyst, oxidant (H_2_O_2_), and sulfur compounds, allowing sufficient duration for oxidation and adsorption processes. For the Ni–Co–Fe composite catalyst (T1), peak efficiency (~ 68%) is achieved at 90 min, as reactive oxygen species (ROS) fully oxidize refractory sulfur molecules into polar sulfones, which are then adsorbed onto the catalyst’s mesopores^70,71^. However, extending the reaction time beyond this optimal duration leads to a decline in sulfur removal (e.g., ~ 40% at 120 min), attributed to two key factors. The first is catalyst saturation, where active sites become occupied by oxidized sulfur species, limiting further adsorption of sulfur compounds. The second factor is partial desorption of sulfones from the catalyst surface due to prolonged ultrasonic cavitation, which produces localized high-energy microenvironments that weaken the interactions between sulfones and the catalyst. Additionally, extended operating time may promote side reactions, such as H_2_O_2_ decomposition or hydrocarbon oxidation, reducing oxidant availability and selectivity^72^. The interplay between reaction kinetics and equilibrium dictates this trend, with shorter times favoring rapid initial oxidation/adsorption and longer times destabilizing the system. Ultrasonic assistance accelerates kinetics early in the process but can also intensify desorption effects and catalyst deactivation. These findings underscore the necessity of optimizing operating time to balance reaction completeness, energy consumption, and catalyst longevity, ensuring efficient sulfur removal without compromising process stability.

#### Impact of catalyst dose change on the level of sulfur removal

**Fig. 13:**
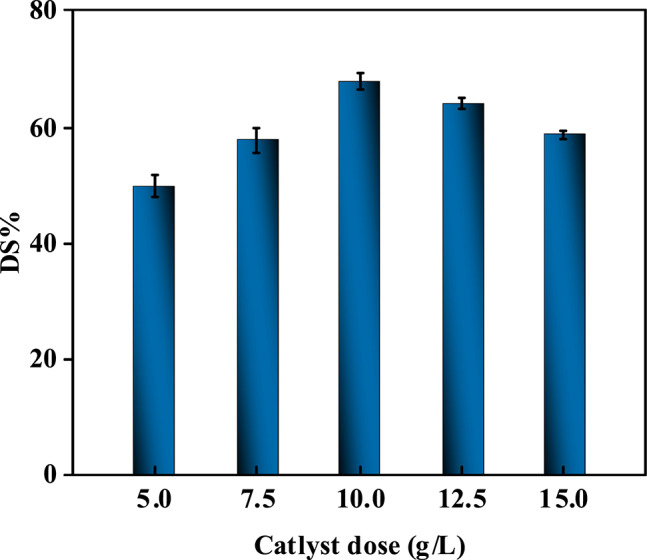
Influence of changing catalyst amounts on the percentage of sulfur removal (at T = 30ºC, H_2_O_2_/Feed = 1:1, t = 1.5h,)

Figure 13 and Table S4 reveal a non-linear relationship between catalyst dosage and sulfur removal efficiency, with an optimal catalyst dose of 10 g/L achieving peak performance (~ 67.7% sulfur removal). At lower doses (5–7.5 g/L), insufficient active sites limit oxidative and adsorptive capacities, resulting in incomplete sulfur oxidation (e.g., ~ 50–60% removal). Increasing the catalyst amount to 10 g/L maximizes surface area accessibility and active site availability, enhancing H₂O₂ activation (via Fe^3^⁺/Co^2^⁺ redox cycles) and sulfur compound adsorption through pore confinement and chemical interactions^70^. However, exceeding this threshold (12.5–15 g/L) induces particle agglomeration, reducing effective surface area and pore accessibility due to interparticle stacking. Aggregates block mesopores, hinder mass transfer, and scatter ultrasonic waves, diminishing cavitation efficiency and ROS accessibility. In addition, oversaturation of active sites can limit further adsorption of sulfur species, while mass-transfer resistance may hinder the diffusion of reactants to catalytic centers. These effects collectively contribute to reduced catalytic efficiency and may promote partial desorption of sulfones rather than their continued oxidation^73,74^. The inverse trend at higher doses underscores the critical balance between active site availability and agglomeration-driven limitations. The study emphasizes that optimal dosing aligns catalyst dispersion, surface reactivity, and ultrasonic synergies, ensuring efficient sulfur removal while minimizing resource waste and operational costs^71^.

#### Effect of reaction temperature on sulfur removal

**Fig. 14:**
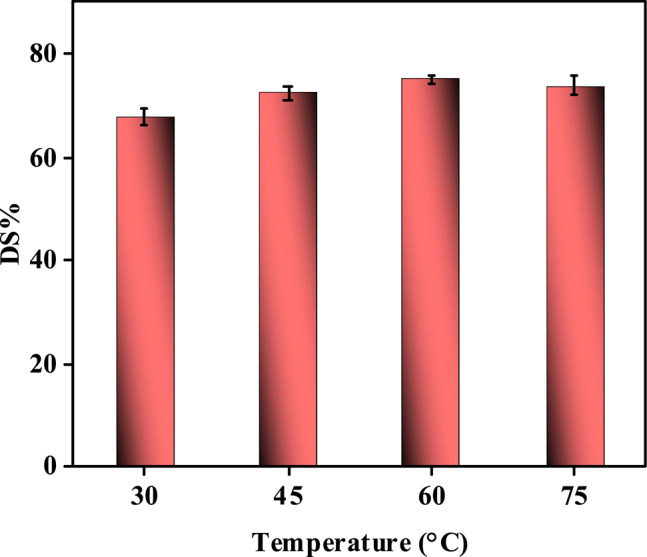
Relationship between operating temperature change and the percentage of sulfur removal (at H_2_O_2_/Feed = 1:1, t = 1.5 h, dose = 10 g/L).

The operating temperature exerts a profound influence on sulfur removal efficiency, governed by the interplay of reaction kinetics, adsorption–desorption equilibrium, and oxidant stability (Fig. 14 and Table S5). At lower temperatures (30–45 °C), sulfur removal efficiency is suboptimal (~ 60–70%) due to sluggish reaction kinetics, where insufficient thermal energy slows the activation of H_2_O_2_ into reactive oxygen species (ROS) and limits the oxidation of refractory sulfur compounds (e.g., dibenzothiophene) into polar sulfones. As temperature increases to 60 °C, efficiency peaks (~ 75%), as thermal energy accelerates Arrhenius-type reaction kinetics, enhancing H_2_O_2_ decomposition into hydroxyl radicals (•OH) and improving the mobility of sulfur molecules to access catalytic sites^71,72^. This temperature optimizes the balance between ROS generation and sulfur adsorption, enabling complete oxidation and subsequent removal via pore confinement. However, exceeding 60 °C (e.g., 75 °C) reverses this trend, reducing efficiency to ~ **73%** due to thermal desorption of weakly adsorbed sulfones from the catalyst surface and H_2_O_2_ decomposition into non-reactive O_2_ and H_2_O, which depletes oxidant availability. Additionally, elevated temperatures may destabilize the catalyst’s structural integrity, promoting agglomeration or phase changes that reduce active site accessibility^75,76^. Ultrasonic cavitation, while enhancing mixing, cannot fully offset these thermal drawbacks at higher temperatures. The study highlights that 60 °C represents the thermodynamic sweet spot, maximizing sulfur oxidation rates while maintaining catalyst stability and oxidant efficiency. This finding could underscore the necessity of precise temperature control to achieve deep desulfurization without triggering adverse thermal effects^77^.

The operating temperature significantly influences sulfur removal efficiency by affecting both the kinetics of oxidative desulfurization (ODS) and the stability of the oxidant. As shown in Fig. 13, sulfur removal efficiency increases from ~ 68% at 30 °C to a maximum of ~ 75% at 60 °C, after which a decline is observed (~ 73% at 75 °C). This trend reflects the balance between enhanced Arrhenius-type kinetics at moderate temperatures and adverse effects such as H₂O₂ decomposition and thermal desorption of sulfones at elevated temperatures.

To quantitatively assess the kinetic contribution, pseudo-first-order rate constants (k) were determined from sulfur concentration data at different temperatures. The resulting Arrhenius plot (ln k vs. 1/T, Figure S1) shows a good linear correlation, from which the apparent activation energy (Ea) was calculated to be 13.7 ± 0.6 kJ·mol⁻^1^. This relatively low Ea highlights the role of ultrasonic cavitation in lowering the energetic barrier by facilitating rapid decomposition of H₂O₂ into reactive oxygen species (ROS) and improving interfacial mass transfer.

The Arrhenius analysis (Figure S1, Electronic supporting information (ESI)) further reveals that sulfur removal follows pseudo-first-order kinetics with an apparent activation energy of ~ 13.7 kJ·mol⁻^1^, which is relatively low compared to conventional oxidative desulfurization. This low barrier reflects the synergistic effect of ultrasonic cavitation and the redox-active Ni–Co–Fe composite, which facilitate efficient H₂O₂ activation into ROS under mild thermal conditions. The rate constant (k) increases from 0.0117 min⁻^1^ at 30 °C to 0.0191 min⁻^1^ at 60 °C, indicating accelerated reaction kinetics with rising temperature in accordance with Arrhenius behavior (Table 3). However, at 75 °C, k decreases to 0.0173 min⁻^1^, attributed to the non-productive decomposition of H₂O₂ into O₂/H₂O and thermal desorption of weakly bound sulfones from the catalyst surface.

**Table 3:** Pseudo-first-order rate constants (k) for sulfur removal at different temperatures.

Temperature (°C)	Temperature (K)	k (min⁻^1^)
30	303.15	0.0117
45	318.15	0.0154
60	333.15	0.0191
75	348.15	0.0173

At the optimum of 60 °C, the system achieves the highest sulfur removal efficiency due to maximized ROS generation and accelerated molecular diffusion, without excessive oxidant decomposition. Beyond this temperature, catalytic sites become less effective as oxidant depletion, sulfone desorption, and competing side reactions dominate, explaining the decline in performance. These findings confirm that 60°C represents the optimal temperature, balancing ROS generation, catalytic activity, and oxidant stability, while higher temperatures compromise efficiency due to thermal and oxidative losses (Table 3).

#### Effect of solvent extraction process on reduction of sulfur content in diesel fuel (Post-UAOD Treatment)

**Fig. 15:**
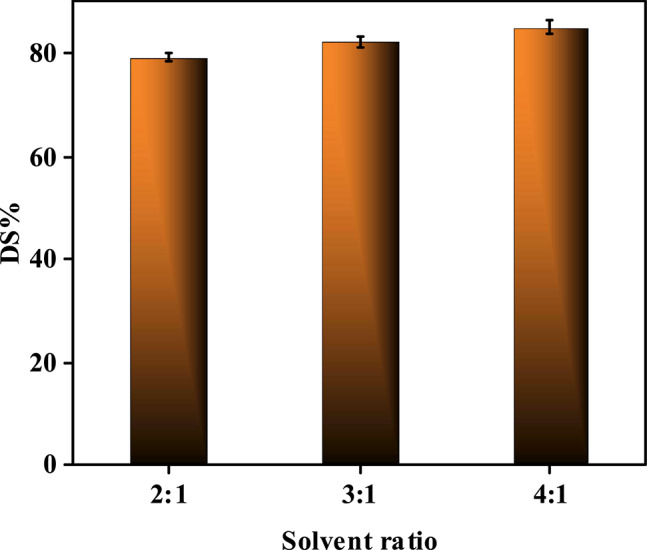
Influence of applying solvent extraction by acetonitrile on the percentage of sulfur removal using the prepared composite (T1) at H_2_O_2_/Feed = 1:1, t = 1.5 h, dose = 10 g/L, T = 60 °C.

The application of acetonitrile as a solvent extraction agent significantly enhances sulfur removal efficiency by selectively isolating oxidized sulfur species (e.g., sulfones) from the diesel feedstock (Fig. 15 and Table S6), complementing the ultrasound-based catalytic oxidative desulfurization. Following oxidation, polar sulfones exhibit higher solubility in polar solvents such as acetonitrile (dielectric constant ~ 37.5) compared to non-polar hydrocarbons, enabling efficient phase separation. The study demonstrates that increasing the acetonitrile-to-feed ratio from 2:1 to 4:1 progressively improves sulfur removal (from ~ 79 to ~ 85%), as higher solvent volumes enhance mass transfer and solvation of sulfones^78,79^. However, beyond a 4:1 ratio, the incremental gain diminishes due to solvent saturation and potential emulsification, which complicates phase separation and increases solvent recovery costs. Acetonitrile’s efficacy stems from its strong hydrogen-bonding capacity and dipole interactions with sulfones, which outcompete weaker van der Waals forces binding sulfones to the hydrocarbon matrix. Additionally, the solvent’s low viscosity and high diffusivity facilitate rapid extraction kinetics, while its miscibility with water aids in post-extraction purification. However, the process faces challenges such as solvent loss, environmental concerns, and energy-intensive recovery, prompting the study to highlight the need for optimizing solvent-to-feed ratios (e.g., 4:1) to balance efficiency and practicality^79^. These findings emphasize solvent polarity and selectivity as key factors in designing sustainable and multi-step desulfurization processes. In a summary, the combined oxidation–extraction strategy achieved as high sulfur removal as 85%,, demonstrating the synergistic role of catalytic pretreatment in improving solvent extraction efficiency.

To accurately understand the provided insights through the post-extraction oxidative desulfurization, it has been necessary to explore the efficiency of direct extraction by acetonitrile (with no pre-oxidation step) on the sulfur removal process. Using the same solvent to feed ratio (4:!}, a sulfur removal percentage of 48.5 was obtained. This finding shows the maximum capability of the solvent to extract sulfur species, highlighting the importance of pre-oxidation which facilitated the extraction process.

#### Influence of solvent type on the desulfurization of diesel fuels after oxidative pretreatment

**Fig. 16:**
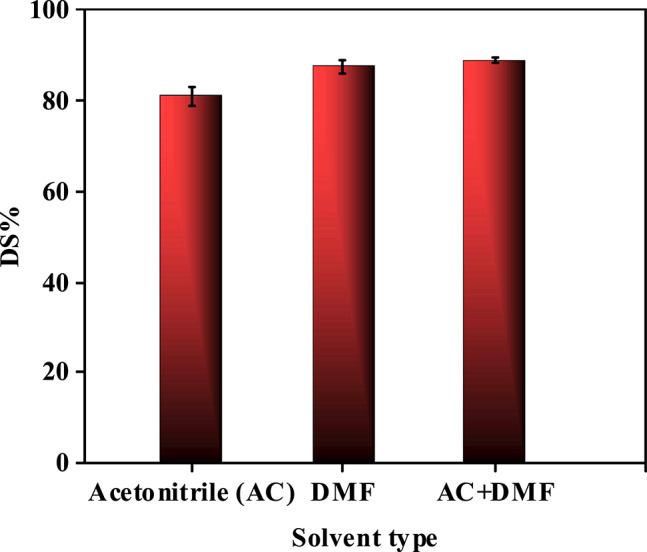
Effect of solvent type on the percentage of sulfur removal using prepared composite T1 (at H2O2/Feed = 1:1, t = 1.5 h, dose = 10 g/L, T = 60 °C, 4:1 solvent-to-feed ratio).

The study demonstrates that solvent type critically influences sulfur removal efficiency (Fig. 16 and Table S7) by dictating the selectivity, solvation capacity, and phase separation dynamics during post-oxidation extraction. Among the tested solvents (acetonitrile, dimethylformamide (DMF), and a DMF-acetonitrile (1:1) mixture), acetonitrile achieves 85% sulfur removal at a 4:1 solvent-to-feed ratio, attributed to its high polarity (dielectric constant ~ 37.5) and strong hydrogen-bonding affinity for polar sulfones. Acetonitrile’s low viscosity and miscibility with oxidized sulfur compounds enable rapid mass transfer and efficient phase separation, minimizing solvent retention in the diesel phase^78,79^. On another side, DMF which has slightly higher polarity (dielectric constant ~ 36.7) and superior solvation power for bulky aromatic sulfones achieved higher efficiency (~ 87.5%) due to its ability to dissolve larger, more complex sulfur derivatives. However, DMF’s higher boiling point (~ 153 °C vs. acetonitrile’s ~ 82 °C) complicates solvent recovery, increasing operational costs and environmental concerns. The DMF-acetonitrile mixture synergizes these properties, achieving 89% sulfur removal by balancing polarity and solvation capacity. This study also highlights the role of solvent selectivity: non-polar solvents (e.g., hexane) perform poorly (< 30% removal) due to weak interactions with polar sulfones, while overly polar solvents (e.g., water) induce emulsification, hindering phase separation. Overall, solvent selection must balance polarity, selectivity, recyclability, and compatibility with catalytic systems to optimize sulfur removal while ensuring economic and ecological viability.

#### Impact of catalyst modification on the sulfur contents of diesel

The modification of the Ni–Co–Fe catalyst (T1) with a polystyrene core–shell structure (TS1) significantly improved sulfur removal performance, lowering the sulfur content in diesel from 21,700 ppm (raw feedstock) to 920 ppm (95.5% removal), compared to 2,810 ppm (87% removal) for the unmodified catalyst (T1). This enhancement arises from the synergistic interplay between the polymer shell and the oxide core, rather than a single dominant factor. The polystyrene provides interactions with aromatic sulfur compounds (e.g., dibenzothiophene), enabling selective adsorption, while the mesoporous oxide core (10 nm pores) preserves pore confinement and metal–sulfur coordination (Fe^3^⁺/Co^2^⁺). Moreover, the hierarchical porosity formed through HIPE polymerization enhances sulfur diffusion, and ultrasonic cavitation minimizes particle agglomeration, maximizing access to active sites. Although the polymer shell slightly reduces magnetization (38 emu/g vs. 45 emu/g for T1), the catalyst maintains sufficient magnetic recoverability for reuse^80^. Beyond structural modification, the aromatic framework of polystyrene mirrors the chemical nature of sulfur-containing aromatics in diesel fuel, creating a "like attracts like" affinity that drives selective uptake of these species^81^. Ultrasonic irradiation further enhances this effect: the polystyrene shell absorbs and redistributes ultrasonic energy, intensifying cavitation. The collapse of cavitation bubbles produces localized hot spots and shock waves that accelerate H₂O₂ activation into reactive oxygen species (•OH), promoting faster sulfur oxidation. Simultaneously, ultrasonication polarizes the polymer’s electronic system, increasing surface polarity and strengthening interactions with polar sulfones^82–86^. This combined action of π–π interactions, cavitation-assisted oxidant activation, and polarity modulation enables the polystyrene-modified catalyst to achieve 95.5% sulfur removal (920 ppm), clearly surpassing the unmodified T1 catalyst (2,810 ppm). The sulfur contents of diesel produced with the catalysts before and after polymer modification are summarized in Table 4.

**Table 4:** Impact of catalyst modification on the sulfur contents of diesel fuels (at T = 60 ºC, H_2_O_2_/Feed = 1:1, t = 1.5h, dose = 10g/L, solvent/feed = 4:1).

Sample	Sulfur content
Feed	21,700
T1 (unmodified catalyst)	2810
TS1 (modified catalyst)	920

### Comparison with other reactor configurations

The performance of the ultrasonic-assisted oxidation–extraction system used in this study was compared with other commonly reported reactor configurations, including Trickle Bed Reactors (TBR), Batch Reactors (BR), Oscillatory Baffled Reactors (OBR), and Double-Baffled Bubble Reactors (DBBR). While TBRs and BRs offer simple operation, they suffer from limited mass transfer rates and longer reaction times. OBR and DBBR improve mixing but still rely on mechanical agitation rather than cavitation. In contrast, the ultrasonic reactor employed in this work generates cavitation microbubbles, leading to localized hotspots and enhanced interfacial contact between immiscible phases, which accelerates oxidative desulfurization and reduces solvent consumption. This synergy resulted in up to 86% sulfur removal at optimized conditions, outperforming most conventional systems reported in literature^87–89^.

## Conclusion

This study demonstrates the successful development of Fe–Co–Ni composites as efficient catalysts for efficient UAOD of diesel. By optimizing operational parameters (60 °C, 90 min, 1:1 H₂O₂-to-feed ratio, and 10 g/L catalyst dose), a sulfur removal efficiency of 55% was achieved, which further increased to 86% via post-extraction process using acetonitrile (as solvent) at a 4:1 solvent-to-feed ratio.. The highest efficiency (95.5%) was obtained using core–shell polystyrene-coated Fe–Co–Ni composites. The superior performance of this hybrid system arises from the combined mechanisms at play: (i) pore confinement effects that facilitate the trapping and oxidation of bulky sulfur molecules, (ii) π–π interactions between the aromatic rings of polystyrene and aromatic sulfur compounds such as DBT derivatives, (iii) metal–sulfur coordination via Fe^3^⁺/Co^2^⁺ redox-active sites, (iv) hydrogen bonding between surface hydroxyl groups and sulfones, and (v) dipole–dipole interactions that increase polarity-driven adsorption. These synergistic mechanisms collectively enhanced both selectivity and catalytic efficiency. Ultrasonic cavitation further amplified desulfurization by generating microbubbles which accelerated reaction kinetics, overcoming traditional mass-transfer limitations in ODS. Overall, this study provides a scalable and energy-efficient strategy for producing low-sulfur diesel. The obtained findings highlight the role of structural design (Fe–Co–Ni composites) alongside with concise of operational parameters to achieve deep desulfurization.

## Supplementary Information

Below is the link to the electronic supplementary material.

Supplementary Material 1

## Data Availability

All the data used will be made available upon reasonable request from the corresponding author.
