# Supplementary material for: Production of low sulfur diesel fuel through ultrasonic catalytic oxidative route using novel mixed oxides nanocomposites assisted by solvent extraction
Source: Sci Rep. 2026 Apr 10;16:12058. doi: 10.1038/s41598-026-39220-0 (PMC13069114; doi:10.1038/s41598-026-39220-0)
Supplement: Supplementary file 1 — Supplementary Material 1 [file 41598_2026_39220_MOESM1_ESM.pdf]

Table S1: Sulfur contents in the produced diesel fuel stocks after treatment by composites (T1-T5).

**Sample**	**Sulfur content**
Feed	21700
T1	9400
T2	10600
T3	10890
T4	11120
T5	11350

Table S2: Effect of H_2O_2***-to-feed ratio on sulfur contents of the produced diesel fuel stocks.***

**Sample**	**Sulfur content**
Feed	21700
1:0.25	10470
1:0.5	10250
1:0.75	9900
1:1	9400
1:1.25	9840

Table S3: Effect of operational times on the process of diesel fuel desulfurization

**Sample**	**Sulfur content**
Feed	21700
0.5h	11800
1h	9400
1.5h	7000
2h	13400

Table S4: Catalyst dose variation versus sulfur contents of gained diesel fuels

**Sample**	**Sulfur content**
Feed	21700
5g	10900
7.5g	9150
10g	7000
12.5g	7800
15g	8950

Table S5: Effect of operational temperature on sulfur contents of produced diesel fuels

**Sample**	**Sulfur content**
Feed	21700
30	7000
45	5990
60	4540
75	5000

Table S6: Effect of solvent-to-diesel fuels ratio on their sulfur contents

**Sample**	**Sulfur content**
Feed	21700
2:1	4540
3:1	3680
4:1	3250

Table S7: Effect of solvent type on the sulfur contents of diesel fuels

**Sample**	**Sulfur content**
Feed	21700
Acetonitrile (AC)	3250
DMF	2970
Acetonitrile + DMF	2810

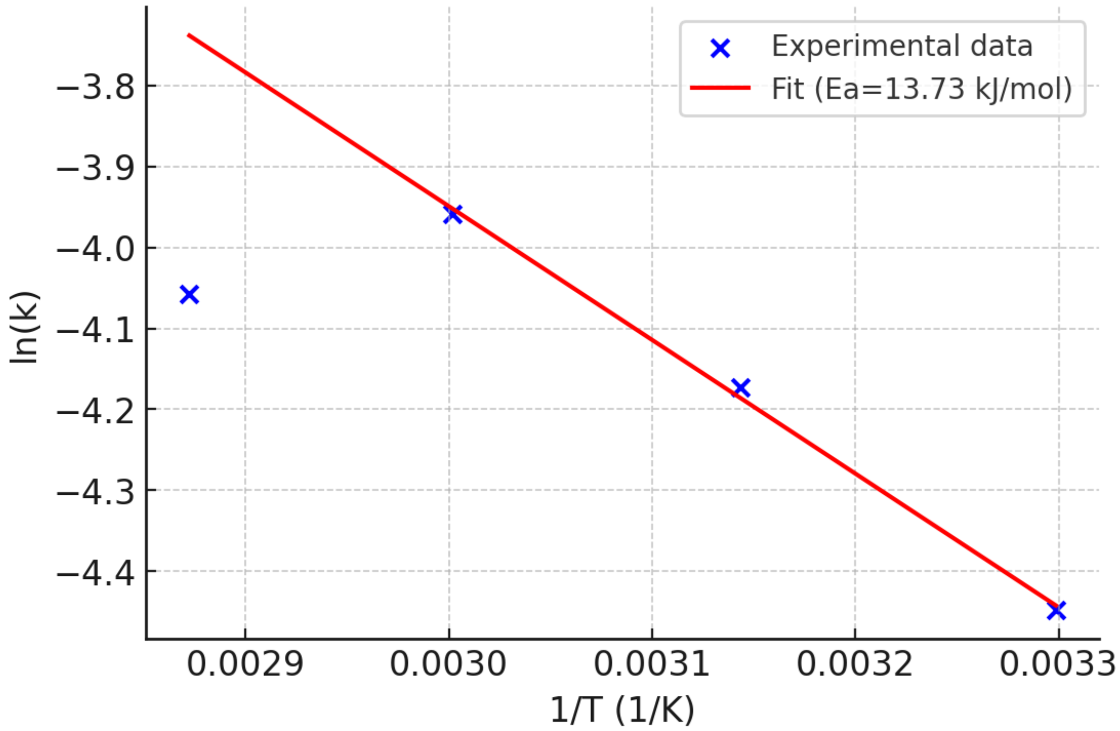

Figure S1: Arrhenius plot (ln k vs. 1/T) for the ultrasound-assisted catalytic oxidative desulfurization process.
